# The effect of AQP4 on tau protein aggregation in neurodegeneration and persistent neuroinflammation after cerebral microinfarcts

**DOI:** 10.1515/med-2023-0800

**Published:** 2023-10-09

**Authors:** Yong Fan, Yongkai Yang, Kunzhe Lin, Xiaohui Zhou, Yongkun Li, Qingqiang Lin

**Affiliations:** Central Laboratory, Affiliated Fuzhou First Hospital of Fujian Medical University, Fuzhou, Fujian, 350009, China; Department of Neurosurgery, Affiliated Fuzhou First Hospital of Fujian Medical University, Fuzhou, Fujian, 350009, China; Department of Neurology, Fujian Provincial Hospital, Shengli Clinical Medical College of Fujian Medical University, No. 134, East Street, Fuzhou, Fujian, 350001, China; College of Life Sciences, Fujian Normal University, Qishan Campus, No. 13 Science and Engineering Building, Fuzhou, Fujian, 350117, China

**Keywords:** cerebral microinfarcts, AQP4, tau protein, neurodegeneration, neuroinflammation

## Abstract

This study aimed to investigate the effect of aquaporin-4 (AQP4) on tau protein aggregation in neurodegeneration and persistent neuroinflammation after cerebral microinfarcts. A model of diffuse ischemic brain injury was established, and adenovirus was injected stereotactically through the lateral ventricle of mice. The water content of the brain tissue was measured. The co-expression of glial fibrillary acidic protein (GFAP) and AQP4 and the aggregation of p-tau and neuronal marker were detected through immunofluorescence double staining. The expression of phosphorylated microtubule-associated protein tau (p-tau, Ser202/Thr205, Thr205, Ser396, Ser404), interleukin(IL)-6, IL-1β, tumor necrosis factor (TNF)-a, growth associated protein43 (GAP43), GFAP, and ionized calcium-binding adapter molecule 1 (Iba1) was detected through Western blot. It was found that the brain water content in the model group was increased and decreased after the AQP4 interference. Compared with the sham group, the expression of GFAP, p-tau, IL-1β, TNF-a, Iba1, and p-tau was increased in the model group (*p* < 0.05). Compared with the model group, the expression of p-tau, IL-6, IL-1β, TNF-a, GFAP, and Iba1 was decreased after AQP4 interference (*p* < 0.05). It is indicated that AQP4 positively regulates neurodegeneration and persistent neuroinflammation caused by tau protein aggregation after cerebral microinfarcts.

## 1 Introduction

Cerebral microinfarct is a common pathological event in patients with dementia and other cerebrovascular diseases. The pathological features of cerebral microinfarcts are consistent with known ischemic infarction such as obliterating vascular disease, embolism, hypoperfusion, and blood–brain barrier disruption [1], which produce persistent brain inflammation and disrupted axon structures in subcortical and cortical tissues, amplifying regional damage and dysfunction [2,3]. Microinfarcts can occur in all brain regions and also lead to neurodegenerative changes in distal brain regions [1,4]. Microinfarcts are a common feature in elderly patients, particularly suffering mild cognitive dysfunction, vascular, or Alzheimer’s disease (AD) [5]. Microinfarcts are reported to lead to vascular dysfunction and further promote AD by impairing cerebral blood flow, exacerbating amyloid β-protein (Aβ) and tau pathology, triggering neuronal death, and inducing glial cell activation [6]. AD is a progressive neurodegenerative disease characterized by neuropathological features associated with the deposition of Aβ plaques and the formation of neurofibrillary tangles associated with tau hyperphosphorylation, leading to brain atrophy with neuronal and synaptic loss [7,8].

Aquaporin-4 (AQP4), the most prevalent aquaporin in the brain, is expressed in all brain regions and plays an important role in hydrohomeostasis and dysregulation of the brain and exerts a pro-inflammatory role by activating microglia and promoting the release of astrocytes from other astrocytes [9]. AQP4 functions as a bidirectional water channel, which facilitates the transfer of water in and out of astrocytes in response to osmotic gradients generated by solute transport [10]. The increased AQP4 activity in cerebral edema is caused by the upregulation of calmodulin at the onset of hypoxia-mediated injury, which binds directly to the AQP4 carboxyl terminus. The large increase in intracellular water leads to cell swelling [11]. Trifluoperazine (TFP) inhibits calmodulin-mediated cell localization of AQP4 by binding directly to calmodulin, which prevents the consequent development of CNS edema. A significant reduction in cerebral edema was observed with subcutaneous injection of TFP 1 h after stroke [12].

In addition, AQP4 acts as a barrier between glymphatic system and brain parenchyma and plays a crucial role in regulating fluid flow between astrocytes, CSF, and blood vessels. AQP4 also mediates glymphatic system to clear Aβ [13]. However, the mechanisms of AQP4 underlying neurodegeneration and persistent neuroinflammation after cerebral microinfarcts are unclear. This study aimed to explore the role and mechanism of AQP4 in neurodegeneration and persistent neuroinflammation and provide potential therapeutic targets for clinical practice of cerebral microinfarct in the future.

## 2 Materials and methods

### 2.1 Materials

ReverTra Ace^®^ qPCR RT Kit (FSQ-301, TOYOBO, Japan); primary antibodies: mouse monoclonal anti-β actin (TA-09, Zhongshan Jinqiao, China, 1/2,000 dilution), rabbit anti-p-tauSer202 (bs-11240R, Bioss, Massachusetts, USA, 1/500 dilution), rabbit anti-p-tauThr205 (bs-5420R, Bioss, Massachusetts, USA, 1/500 dilution), rabbit anti-p-tauThr231 (bs-2368R, Bioss, Massachusetts, USA, 1/500 dilution), rabbit anti-p-tauSer396 (bs-3446R, Bioss, Massachusetts, USA, 1/500 dilution), rabbit anti-p-tauSer404 (bs-2392R, Bioss, Massachusetts, USA, 1/500 dilution), rabbit anti-IL-6 (DF6087, Affinity Biosciences, Cincinnati, OH, USA, 1/500 dilution), rabbit anti-IL-1β (16806-1-AP, Proteintech Group, Inc., IL, China, 1/500 dilution), mouse anti-TNF-a (60291-1-Ig, Proteintech Group, Inc., IL, China, 1/500 dilution), rabbit anti-GFAP (BF0345, Affinity Biosciences, Cincinnati, OH, USA, 1/500), rabbit anti-Iba1 (DF6442, Affinity Biosciences, Cincinnati, OH, USA, 1/500 dilution), rabbit anti-AQP4 (16473-1-AP, Proteintech Group, Inc., IL, China, 1/500 dilution), and rabbit anti-NeuN (26975-1-AP, Proteintech Group, Inc., IL, China, 1/500 dilution); secondary antibodies: horseradish peroxidase (HRP)-labeled goat anti-mouse IgG (H + L) (ZB-2305, Zhongshan Jinqiao, China, 1/2,000 dilution), HRP-labeled goat anti-rabbit IgG (H + L) (ZB-2301, Zhongshan Jinqiao, China, 1/2,000 dilution), goat anti-rabbit IgG cy3 (AS007, ABdonal Technology Co., Ltd., USA, 1/2,000 dilution), and goat anti-mouse IgG/488 (ZF-0511, Zhongshan Jinqiao, China, 1/2,000 dilution).

### 2.2 Mouse treatment and intervention

A total of 42 male C57BL/6 mice (6–8 weeks old, License number: SCXK (Beijing) 2019-0010) were purchased from Spaifu (Beijing) Biotechnology Co., Ltd., China, and bred under the environment of temperature 20–26°C, humidity 40–70%.

The C57BL/6 mice were divided into 6 groups, including: (1) sham surgery treatment group (control, *n* = 7), (2) sham surgery treatment + AQP4 gene adenovirus interference no-load treatment group (control + si-NC, *n* = 7), (3) sham surgery treatment + AQP4 gene adenovirus interference vector treatment group (control + si-AQP4, *n* = 7), (4) brain injury modeling (model, *n* = 7), (5) brain injury modeling + AQP4 gene adenovirus interference no-load treatment group (model + si-NC, *n* = 7), and (6) brain injury modeling + AQP4 gene adenovirus interference vector treatment group (model + si-AQP4, *n* = 7).

### 2.3 Adenovirus injection

The anesthetized mice lied on the brain stereotaxostat after intraperitoneal injection, the head was shaved, disinfected, and the scalp cut to expose the skull. A 10 μL microsampler was used to locate the lateral ventricle, 0.22 mm after the fontanelle, 10 mm side, drilled with 2.3 mm depth starting from the surface of skull. Adenovirus was injected 1 μL/min for a total of 6 μL, after reaching the lateral ventricle. The next step of the experiment was carried out after 4 days.

### 2.4 Brain injury modeling of mice

The procedure of brain injury modeling of mice was conducted as follows. The mice were anesthetized after intraperitoneal injection, lied back, and fixed on the operating table. Mice were anesthetized with pentobarbital sodium (4% w/v) via intraperitoneal injection, an incision in the middle of neck skin was made to expose the subcutaneous tissue and muscle, The right common carotid artery (CCA), internal carotid artery (ICA), and external carotid artery (ECA) were carefully isolated under a microscope. The distal end of the common carotid artery was ligated with a 5-0 suture. The external carotid artery was temporarily clamped with sutures, and the internal carotid artery was temporarily ligated by arterial clamp, while the proximal end of the common carotid artery was also temporarily ligated. An insulin syringe was inserted into the common carotid artery, the internal carotid artery hemostatic clamp was loosened, Then, 3000 ± 500 cholesterol crystals in 100 ul of saline or 100 μl of saline alone (for sham animals) were injected via insulin syringe. After injection, the proximal ECA was permanently ligated and the wound was closed. Mice were monitored after induction of injury.

### 2.5 Measurement of brain water content

One mouse from each group was sacrificed 24 h after microinfarct injury. The brain was obtained and weighed immediately. Then, the brain was put in a 70°C oven for 48 h, weighed, and then dried for another 24 h until reached a consistent weight. The brain water content was calculated according to the following formula: water content% = (brain wet weight − brain dry weight)/brain wet weight × 100%.

#### 2.5.1 Real-time fluorescence quantification polymerase chain reaction (qRT-PCR)

Samples in brain tissue were collected, adding Trizon lysate, and total RNA was extracted. RNA was synthesized by reverse transcription ReverTra Ace^®^ qPCR RT Kit to synthesize cDNA. Real-time fluorescence quantification PCR was performed with the following reaction system: 5 μL 2× Real-time PCR Master Mix, 0.4 μL each F/R Primer (10 μM), 1 μL cDNA Template (1:100 dilution), and 10 μL ddH_2_O. The response steps included: 95°C, 180 s, 40 cycles of 94°C, 15 s; 60°C, 30 s; 72°C, 30 s; and then 95°C, 10 s; 65°C, 60 s; 97°C, 1 s. Glyceraldehyde-3-phosphate dehydrogenase (GAPDH) was used as an internal reference, and the relative expression was calculated according to the 2^−△△Ct^ method. The primers were designed as follows: m-AQP4-Q, 5′-TCAGCATCGCTAAGTCCG-3′ (forward), 5′-CTCCCAATCCTCCAACCA-3′ (reverse); hGAPDH-Q, 5′-TCAAGGCTGAGAACGGGAAG-3′ (forward), 5′-TCGCCCCACTTGATTTTGGA-3′ (reverse).

### 2.6 Western blot (WB)

The total protein of brain tissues was extracted after adding the corresponding lysate and measured using the BCA kit. After protein denaturation, it was put on sodium dodecyl benzenesulfonate gel electrophoresis for 1–2 h and wet transferred to the membrane for 30–50 min. The membrane was incubated with the primary antibodies against GFAP, AQP4, IL-6, IL-1β, TNF-a, p-tau (Ser202/Thr205, Thr205, Thr231, Ser396, Ser404), or Iba1 solution at 4°C overnight and further incubated with the secondary antibody at room temperature for 1–2 h. Finally, enhanced chemiluminescence exposure solution was added to the membrane to expose in the gel imaging system, and the grayscale values of each antibody band were analyzed using the “Quantityone” software (Bio-Rad, CA, USA).

### 2.7 Immunofluorescence detection

At 24h after injury, multiple microinfarct treated or sham-treated mice were perfusion fixed with 4% PFA, brains were removed and postfixed for 24 h, and 10um coronal vibratome sections were cut. Sections were washed and blocked with 5% bovine serum albumin (BSA) with 0.01% Triton X-100. Slices were incubated with the following primary antibodies: anti-GFAP (1:200 dilution), anti-P-tau (1:200 dilution), anti-AQP4 (1:200 dilution), anti-NeuN (1:200dilution), 4°C incubation overnight. Secondary detection was performed with Cy3 (1:2,000 dilution) and IgG/ 488 (1:100 dilution) at 37°C for 3 h. Slices were mounted with DAPI (Invitrogen) to stain nuclei. Image-Pro Plus image analysis software (Media Cybernetics, USA) was used to measure the average optical density value of immunofluorescence staining pictures and perform a semi-quantitative analysis of protein co-localization for each group.

### 2.8 Statistical analysis

Statistical Product Service Solutions (SPSS) version 20.0 software (SPSS Inc., Chicago, IL, USA) was used for statistical analysis. All experiments were repeated 3 times. Quantitative results were presented with mean ± standard deviation. The quantitative numerical comparison between the two groups was conducted by independent sample *T* test. The comparison between multiple groups was done by one-way analysis of variance, with the least significant difference method used for each two-group comparison. *p* < 0.05 indicated a significant difference.


**Ethical approval:** All procedures contributing to this work comply with the ethical standards of the relevant national guides on the care and use of laboratory animals and have been approved by the Ethics Committee of Fujian Medical University (IACUC FJMU 2022-NSFC-0318).

## 3 Results

### 3.1 Effects of AQP4 on GFAP and p-tau aggregation

**Figure 1:**
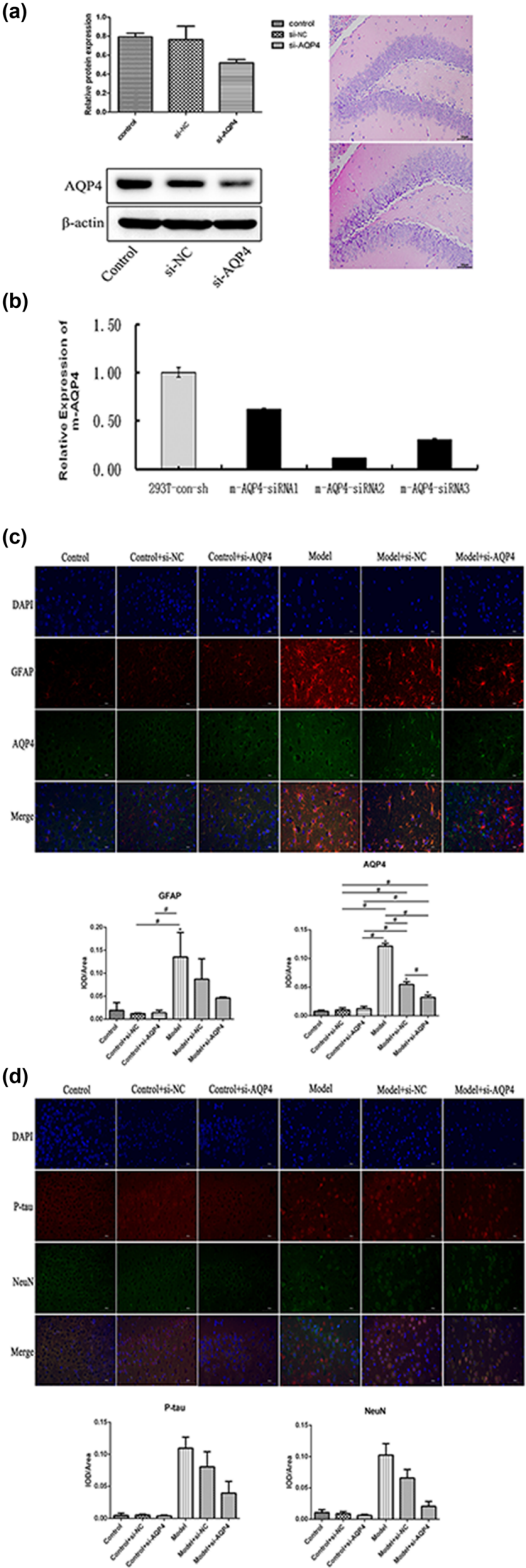
Effects of AQP4 on GFAP and p-tau aggregation. (a and b) Histology, qPCR, and Western blot assays verify adenovirus efficiency; (c and d) GFAP and AQP4 and p-tau aggregation (pSer202/pThr205, p-tau) and neuronal marker (NeuN) co-expression were evaluated by Immunofluorescence double staining. *p < 0.05 vs control. All experiments were repeated 3 times. Each group has 7 mice.

From Figure 1a, it is clear thatAQP4 expression levels were detected by qRT-PCR and WB to verify adenovirus efficiency, and the results showed that AQP4 mRNA and protein expression was downregulated, indicating that the interference was effective. The water content measurement is shown in Table 1. Compared with the sham group, the brain water content of the model group was increased and then decreased after the AQP4 interference (*p* < 0.05). The levels of P-tau, AQP4, Iba1, and GFAP were elevated in brain injury (*p* < 0.05). The results of immunofluorescence double staining for the detection of GFAP and AQP4 co-expression, as well as p-tau (pSer202/pThr205) aggregation and neuronal marker (NeuN) (Figure 1c, 1d), showed that, compared with the sham group, GFAP and p-tau were increased in the model group and decreased after AQP4 interference.

**Table 1:** Determination of cerebral water content

Groups	Brain wet weight	Brain dry weight	Total water content (%)
Control	0.337	0.078	77.01
Control + si-NC	0.306	0.065	78.75
Control + si-AQP4	0.356	0.072	79.78
Model	0.420	0.061	85.58^*^
Model + si-NC	0.351	0.050	85.77
Model + si-AQP4	0.361	0.064	82.27^#^

Note: *p < 0.05 vs control, ^#^ *p* < 0.05 vs model.

### 3.2 Effects of AQP4 on neuroinflammation

**Figure 2:**
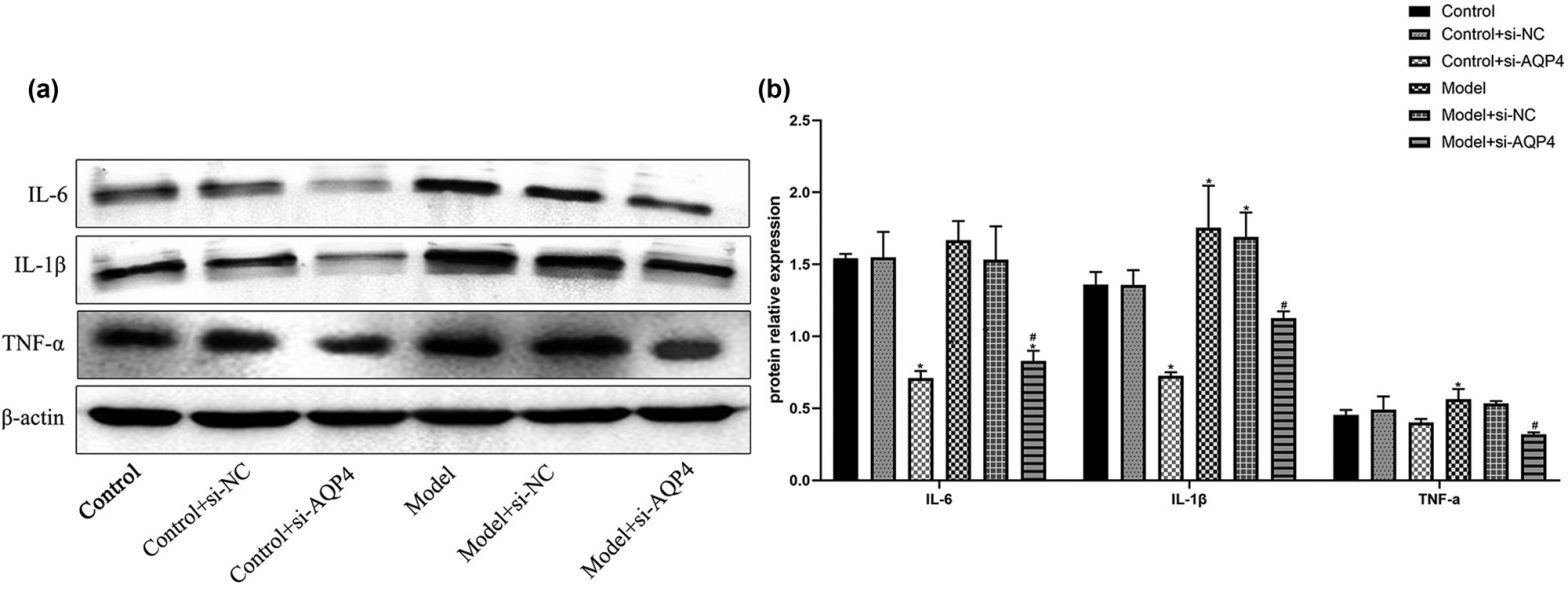
Effects of AQP4 on neuroinflammation. The expression of IL-6, IL-1β, and TNF-a in brain tissue was detected in Western blot assay. *p < 0.05 vs control, ^#^ *p* < 0.05 vs model. All experiments were repeated 3 times. Each group has 7 mice.

The expression of brain tissue inflammation factors IL-6, IL-1β, and TNF-a was detected by WB (Figure 2a). The results showed that compared with the sham group, the expression of IL-1β and TNF-a in the model group was significantly increased (*p* < 0.05; Figure 2b), and the expression of IL-6 was also increased. Compared with the model group, the expression of IL-6, IL-1β, and TNF-a in the model + si-AQP4 group was significantly reduced (*p* < 0.05; Figure 2b). The results indicated that the interference of AQP4 expression can improve the inflammatory response after cerebral microinfarcts.

#### 3.2.1 Effect of AQP4 on the expression of GFAP protein in astrocytes and Iba1 protein in microglia

**Figure 3:**
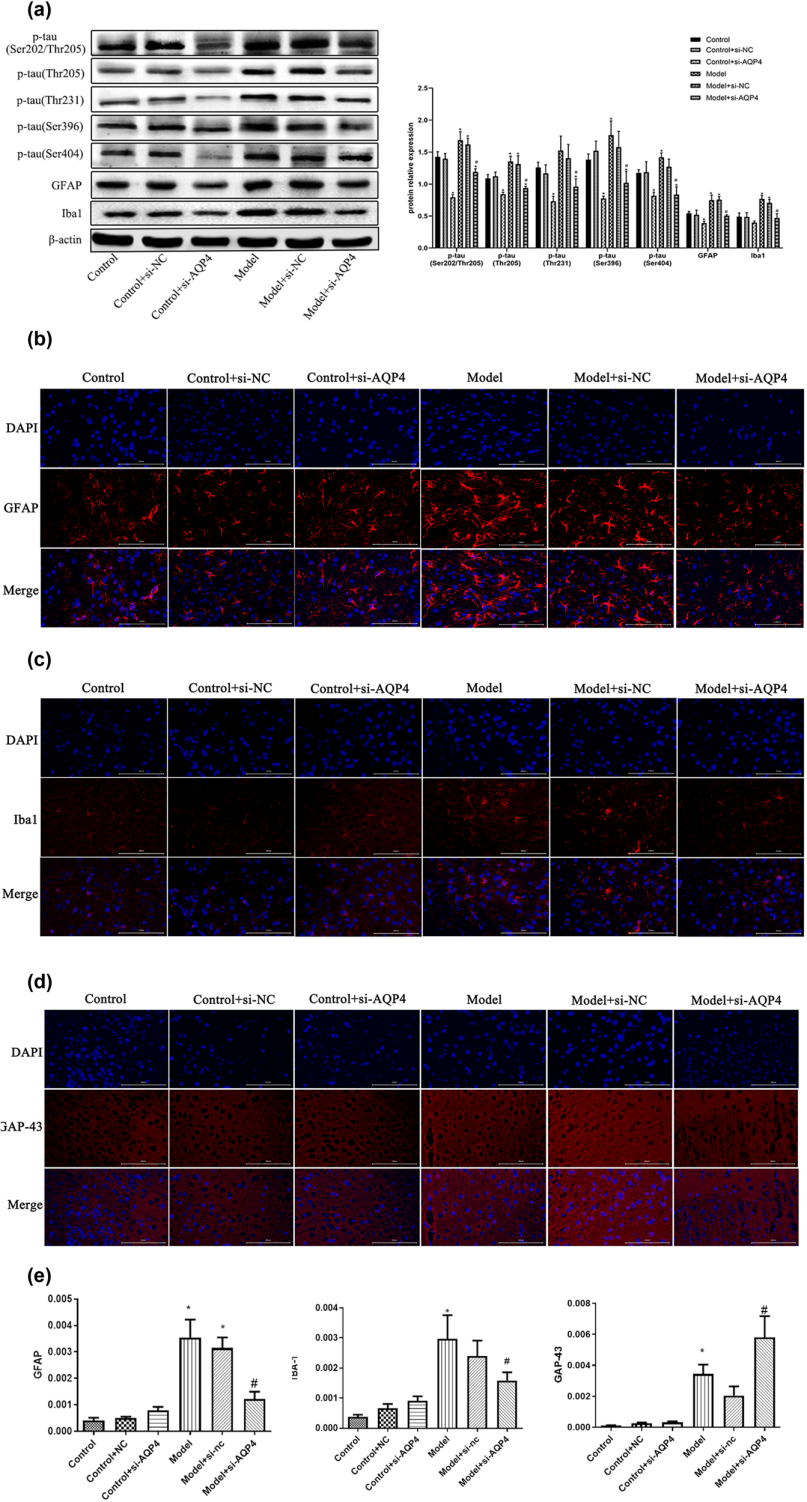
Effects of AQP4 on astrocytes, microglia, and neurodegeneration. (a) The expression of p-tau (Ser202/Thr205, Thr205, Thr231, Ser396, Ser404) and Iba1 in brain tissue was detected by Western blot. (b–e) GFAP, iba1 and GAP43 were detected by Immunofluorescence double staining and analyzed results were obtained (e). *p < 0.05 vs control, ^#^ *p* < 0.05 vs model. All experiments were repeated 3 times. Each group has 7 mice.

The expression of p-tau (Ser202/Thr205, Thr205, Thr231, Ser396, Ser404), GFAP in responsive astrocytoplasia, and Iba1 in microglia in brain tissue was detected by WB and shown in Figure 3a. It was indicated that, compared with the sham group, the expression of p-tau (Ser202/Thr205, Thr205, Ser396, Ser404), GFAP, and Iba1 in the model group was significantly increased (*p* < 0.05), and the expression of p-tau (Thr231) was also increased without significance (*p* > 0.05). Compared with the model group, the expression of p-tau (Ser202/Thr205, Thr205, Ser396, Ser404, Thr231) and the expression of GFAP and Iba1 in the model + si-AQP4 group were significantly reduced (*p* < 0.05). The expression of GAP43, Iba1, and GFAP was further measured by immunofluorescence, as shown in Figure 3b–3e. The results showed that the expression of Iba1 and GFAP in the model group was significantly increased, compared with the sham group (*p* < 0.05). The expression of Iba1 and GFAP was significantly lower (*p* < 0.05) in the model + si-AQP4 group compared to the model group, while the expression of GAP43 was significantly higher (*p* < 0.05) compared to the sham group. The results indicated that although the low expression level of these two genes in the sham group was not achieved by si-AQP4 intervention, the expression of Iba1 and GFAP in the model group was significantly decreased, and AQP4 had negative feedback regulation with Iba1 and GFAP, respectively.

## 4 Discussion

In this study, we explored the potential effects of AQP4 on neurodegeneration and persistent neuroinflammation in a mouse model of diffuse cerebral microinfarcts. AQP4 interference adenovirus was injected stereotactically into the lateral ventricle to examine the effect of decreased AQP4 expression on tau protein aggregation and persistent neuroinflammation and the association with microinfarct tissue damage.

During the course of the study, it was found that the high expression of AQP4 in the brain injury model was able to successfully reduce the protein expression of Iba1 and GFAP by intervening with si-AQP4 in the model group. It is worth noting that the model group was also able to influence the expression of the p-Tau protein to be down-regulated at the same time (although still higher than that of the sham surgery treatment group), which may be able to slow down the brain injury to some extent and is an important early driver of neurodegeneration as described in the study of p-Tau by Kim et al. [14]. In addition, the water content and the expression level of GFAP, p-tau, IL-6, IL-1β, and TNF-a were decreased after AQP4 interference.

Although microinfarction accounts for only a small fraction of the total brain volume, the volume of functionally deficient cortical tissue may be up to 12 times the volume of the microinfarct core due to the impaired hemodynamic responses of the peri-infarct tissue, neurovascular uncoupling, and disruption of neuronal circuits [15]. In addition, the occurrence of microinfarcts is associated with rapid progression of dementia and deterioration of cognitive function [16–18]. The presence of reactive astrocytes in the peri-infarct area of cerebral microinfarcted mice alleviates ischemic brain injury and AD through anti-inflammatory effects [19]. Glial cell-associated inflammation was strongly associated with Aβ expression, while the accumulation of Aβ in the brain triggered microglial activation, which plays a dual role in AD pathology, promoting early neuronal protection by clearing Aβ, but promoting neuronal loss in advanced stages by releasing neurotoxic and pro-inflammatory factors [20–23]. Microinfarcts destruct the integrity of microvascular and microstructural tissue, leading to Aβ deposition and tau phosphorylation, which can increase the risk of dementia, with local edema, acute astrocyte loss, neuronal death, impaired pericytic, and vascular coverage [1]. The accumulation of reactive astrocytes expressed GFAP around the infarct to induce glial scarring in the cerebral cortex [24]. Moreover, the use of nonselective nonsteroidal anti-inflammatory drugs (NSAIDs) is associated with a reduced risk of AD [25]. Previous evidence showed that the accumulation of Aβ and hyperphosphorylated tau in the brains of AD model mice is reduced after being treated with NSAIDs [26–28]. Higher levels of Aβ and tau are found to exacerbate the effects of vascular lesions on ischemic tissue lesions, and higher tau may be associated with a greater risk of microinfarcts in the subcortical region [29].

Research on the direct or indirect involvement of AQP4 in the pathogenesis of microinfarcts is very limited. However, studies have shown that the elevated expression of AQP4 following spinal cord compression injury can lead to spinal cord edema (tissue swelling) and excessive neuronal death, ultimately exacerbating neurological deficits, all of which are partially reversed in AQP4-deficient mice [30]. Inhibition of AQP4 expression levels can alleviate blood–brain barrier destruction and astrocytes edema in traumatic brain injury mice, while restoring AQP4 localization at the end of perivascular astrocytes can protect the integrity of the blood–brain barrier [31]. In addition, Venkat et al. found that the expression of AQP4 was decreased and glymphatic dysfunction was induced with a multiple microinfarction model in retired breeder rats [32]. Upregulating the expression of GAP43 can reduce infarct volume and promote nerve function recovery and nerve fiber regeneration [33]. GAP43, a molecular marker of neuronal development and plasticity, plays a key role in guiding axon growth, regulating axon formation of new connections, and maintaining synaptic function and transmitter release. The expression of GAP43 is greatly increased at the early stage of ischemia [34,35]. Previous studies have provided the evidence that GAP43 is associated with neurodegeneration or neuroinflammation [36,37]. Furthermore, in this study, it is found that the expression of GFAP and p-tau is increased, leading to a further higher expression of inflammatory indicators of IL-6, IL-1β, and TNF-a in the diffuse cerebral microinfarcts model. And the effects can be reversed by AQP4 interference. Based on the above results, AQP4 affecting tau protein aggregation is associated with the nerve degeneration and persistent neuroinflammation-related signaling pathways in cerebral microinfarcts. Further evidence is needed to verify the findings and further explore the potential therapeutic target for cerebral microinfarcts.

There are still several limitations in this study: first, the number size of samples was limited. Although these observations suggest that AQP4 is associated with tau aggregation in neurodegeneration and persistent neuroinflammation after cerebral microinfarcts, more detailed analyses in more patients or experimental animal models are needed. Second, pathological hallmarks of AD include the formation of extracellular Aβ protein and p-tau protein. We only evaluated the changes in p-tau but the important factor Aβ was not investigated.

In summary, our results suggested that AQP4 signaling pathway activation might be associated with tau aggregation in neurodegeneration and persistent neuroinflammation after cerebral microinfarcts. This finding may provide new evidence on the association between AQP4 and tau protein aggregation in neurodegeneration and persistent neuroinflammation, which may help improve the therapeutic intervention of cerebral microinfarcts in the future.

## Supplementary Material

Supplementary Figure
