# Supplementary material for: The effect of AQP4 on tau protein aggregation in neurodegeneration and persistent neuroinflammation after cerebral microinfarcts
Source: Open Med (Wars). 2023 Oct 9;18(1):20230800. doi: 10.1515/med-2023-0800 (PMC10590608; doi:10.1515/med-2023-0800)
Supplement: Supplementary Figure [file med-2023-0800-sm.pdf]

Supplementary material

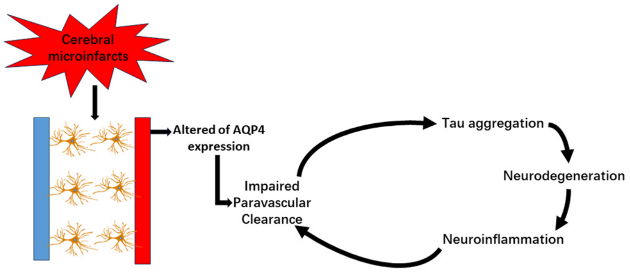

Figure S1: Schematic representation of proposed relationship between altered of AQP4 expression, tau aggregation, neurodegeneration, and persistent neuroinflammation after cerebral microinfarcts.
